# A Microfluidic Platform Revealing Interactions between Leukocytes and Cancer Cells on Topographic Micropatterns

**DOI:** 10.3390/bios12110963

**Published:** 2022-11-02

**Authors:** Xin Cui, Lelin Liu, Jiyu Li, Yi Liu, Ya Liu, Dinglong Hu, Ruolin Zhang, Siping Huang, Zhongning Jiang, Yuchao Wang, Yun Qu, Stella W. Pang, Raymond H. W. Lam

**Affiliations:** 1 Key Laboratory of Biomaterials of Guangdong Higher Education Institutes, Department of Biomedical Engineering, Jinan University, Guangzhou 519070, China; 2 Department of Biomedical Engineering, City University of Hong Kong, Hong Kong 999077, China; 3 Research Center of Biological Computation, Zhejiang Laboratory, Hangzhou 311100, China; 4 BGI-Shenzhen, Shenzhen 518083, China; 5 Department of Electrical Engineering, City University of Hong Kong, Hong Kong 999077, China; 6 Department of Biomedical Engineering, The Hong Kong Polytechnic University, Hong Kong 999077, China; 7 Centre for Biosystems, Neuroscience, and Nanotechnology, City University of Hong Kong, Hong Kong 999077, China; 8 Centre for Robotics and Automation, City University of Hong Kong, Hong Kong 999077, China; 9 Shenzhen Research Institute, City University of Hong Kong, Shenzhen 518057, China

**Keywords:** live-cell immunoassay, topographic micropatterns, on-chip cytokine detection, nasopharyngeal cancer, integrated microfluidics

## Abstract

Immunoassay for detailed analysis of immune−cancer intercellular interactions can achieve more promising diagnosis and treatment strategies for cancers including nasopharyngeal cancer (NPC). In this study, we report a microfluidic live−cell immunoassay integrated with a microtopographic environment to meet the rising demand for monitoring intercellular interactions in different tumor microenvironments. The developed assay allows: (1) coculture of immune cells and cancer cells on tunable (flat or micrograting) substrates, (2) simultaneous detection of different cytokines in a wide working range of 5–5000 pg/mL, and (3) investigation of migration behaviors of mono- and co-cultured cells on flat/grating platforms for revealing the topography-induced intercellular and cytokine responses. Cytokine monitoring was achieved on-chip by implementing a sensitive and selective microbead-based sandwich assay with an antibody on microbeads, target cytokines, and the matching fluorescent-conjugated detection antibody in an array of active peristaltic mixer-assisted cytokine detection microchambers. Moreover, this immunoassay requires a low sample volume down to 0.5 μL and short assay time (30 min) for on-chip cytokine quantifications. We validated the biocompatibility of the co-culture strategy between immune cells and NPC cells and compared the different immunological states of undifferentiated THP-1 monocytic cells or PMA-differentiated THP-1 macrophages co-culturing with NP460 and NPC43 on topographical and planar substrates, respectively. Hence, the integrated microfluidic platform provides an efficient, broad-range and precise on-chip cytokine detection approach, eliminates the manual sampling procedures and allows on-chip continuous cytokine monitoring without perturbing intercellular microenvironments on different topographical ECM substrates, which has the potential of providing clinical significance in early immune diagnosis, personalized immunotherapy, and precision medicine.

## 1. Introduction

Nasopharyngeal carcinoma (NPC) is a major health problem for the Southeastern Asian and North African populations [1,2,3], which can be divided into Epstein-Barr virus (EBV)-positive and negative subgroups. Although current treatments, including radiotherapy, chemotherapy or chemo-radiotherapy, can improve the survival rates, treatment resistance and tumor recurrence remains to date challenging, largely because of the unique and complex intercellular interactions between cancer cells and immune cells, involving various soluble factors released by the tumor microenvironment. The presence of EBVs can alter the biomolecular secretion of NPC cells as well as the immune responses to them. The non-keratinizing Epstein-Barr virus (EBV)-positive nasopharyngeal carcinoma represents a unique tumor microenvironment, characterized by dense infiltrating immune cells comprising macrophages [1]. For instance, EBV-infected NPC cells secreted a higher level of IL-1α (1561 pg/mL), IL-1β (16.6 pg/mL) and IL-8 (422.9 pg/mL) as compared to EBV-negative cells [4]. Macrophages secrete cytokines such as TNF (tumor necrosis factor), IL-1, IL-6, IL-8, and IL-12, while the stimulated ones produce more TNF-α, IL-12/IL-23p40, and IL-10 [5,6]. In addition, EBV-infected human monocytes induce the expression of MCP-1 (monocyte chemotactic protein-1) via Toll-like receptor (TLR) 2 [7]; and therefore such MCP-1 further inhibits the IL-12 production by inflammatory macrophages [8]. Clinical studies also highlighted that high serum levels of MCP-1, TNF-α, IL-6 and IL-8 are the most prominent cytokines associated with bone invasion, distant metastasis, and particularly poor outcome in NPC patients [1,7,9]. Hence, precisely investigating and quantifying intercellular interactions and cytokine secretions from immune and cancer cells would help in understanding the NPC developments and identifying potential targets for optimized NPC diagnose and treatments.

The enzyme-linked immunosorbent assay (ELISA) is widely used as a “gold standard” method for cytokine quantifications, which rely on repeated time-consuming sample incubation and washing procedures and cannot be applied for in-situ, real-time and multiplex cytokine profiling. Over the past few decades, microfluidic-based immunoassays have been developed for rapid analysis of cytokine secretion in complex fluidic bio-samples due to the significant advantages of microfluidics in fluid flow controlling and ultra-low reagent consumption [10,11,12,13]. For example, a strategy reported by Han et al. is capable of multidimensionally analyzing single cells cytokine secretion frequencies by quantitative micro-engraving [14]. Baraket et al. designed an integrated electrochemical biosensor platform which can perform highly sensitive multi-detection for IL-10 and IL-1β [15]. Choi et al. designed a microfluidic magnetic-beads-based device for protein analysis and bio-molecule detection [16]. Min et al. also developed a microfluidic immunoassay platform for biomolecular quantitative detection, which was based on acridine esterification chemiluminescence [17]. To illustrate the dynamic cytokine profiles of various immune cell subtypes, Junkin et al. developed an automated high-throughput microfluidic chip to rebuild the dynamics of single immune cell [18], which was only supposed to detect the cytokine secretion of single types of immune cells. To achieve the multiple cytokine profiling, our pervious study developed an automated microfluidic microbeads-based device for dynamic immunoassay, which profiled multiple cytokines secretion with a low detection limit and short testing time [19]. However, cells growing in regular culture wells cannot reflect the three-dimensional extracellular biochemical and morphologic environments and more physiological-relevant results [20,21]. Our previous studies showed that nasopharyngeal carcinoma cell migration dynamics and spreading directionality can be regulated by microenvironmental morphology [22], suggesting that the grating-like topography of pterygoid muscles can play a role in nasopharyngeal cancer spreading [23]. Andersson et al. demonstrated the effects of substrate morphology on epithelial cell morphologic behaviors and cytokine secretions [24]. Furthermore, the direct observation of cell behaviors such as migration, together with cytokine measurement, may facilitate the future development of new prognostic tools to reveal cancer-immune cell interactions.

Herein, we report a microfluidic immunoassay device by integrating the cell culture region with microtopographic substrates and our previously reported cytokine dynamics profiling scheme [25,26] by implementing a microbeads-based immunofluorescence assay for achieving more sensitive and parallel detection of multiple cytokines. Co-cultured immune cells (undifferentiated THP-1 monocytic cells or PMA-differentiated THP-1 macrophages) and immortal cells NP460/NPC43 were cultured in the device, in which cell culture medium was extracted from the cell culture at different time points, transferred and analyzed in the cytokine detection microchambers for quantifying secretions of TNF and IL-12p70 throughout the co-culture period. As IL-12p70 secretion of monocytes can reflect their EBV infection, these results can quantitatively reflect the role of EBV in immune responses upon nasopharyngeal carcinoma.

## 2. Materials and Methods

### 2.1. Device Fabrication

**Figure 1:**
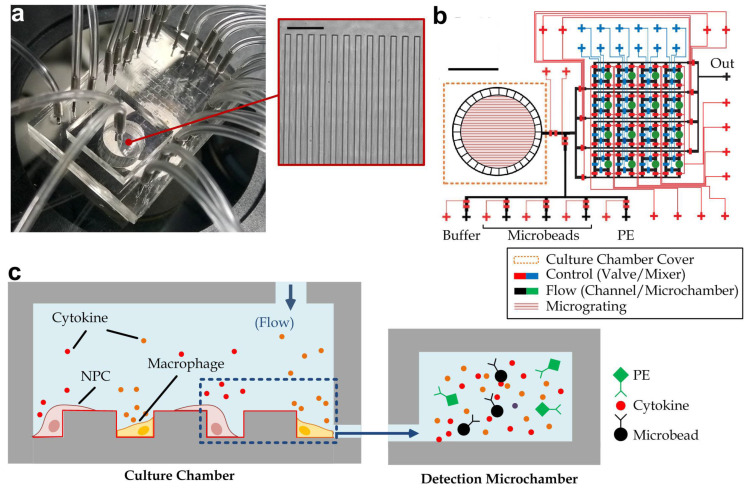
(**a**) Representative microscopic image of a microfluidic immunoassay device integrated with an ECM protein-coated topologic environment. Inset: microscopic image of the parallel grating array in cell culture chamber (grating width: 18 μm, the depth: 18 μm). Scale bar: 100 µm. (**b**) The integrated microfluidic immunoassay device microchannels in the control layer consist of two functional units: valves (*cyan*) for micromixers and valves (*red*) for flow control. The flow channels are composed of an array of microchambers (*green*) for microbead trapping, connecting channels (*black*) between the cell culture region and the downstream cytokine detection arrays. Specially, the connecting microchannels surrounding cell culture chamber contained multiple micro-gaps (height 4 µm; width 50 µm) to allow liquid flow while simultaneously confining the cells in the culture chamber. Scale bar: 4 mm. (**c**) Schematic of the integrated microfluidic immunoassay device operation. Immune cells (macrophages) and nasopharyngeal cells (NPC) were co-cultured in the grating array-embedded cell culture chamber, and the media with secreted cytokines from the cell culture chamber were extracted and incubated with pre-loaded antibody-conjugated microbeads for capturing cytokines in one of the cytokine detection-microchamber. Fluorescent-conjugated detection antibodies were used to label the captured cytokine for microscopic imaging-based multiple cytokine quantification.

The reported microfluidic device (Figure 1a) consists of three layers of microstructures made of polydimethylsiloxane (PDMS; Dow Corning, Midland, MI, USA): a control layer (height: 20 μm), a flow layer (gas channel height: 20 μm) and a micro−grating layer, with the design layout as illustrated in Figure 1b. The device was fabricated by multilayer soft lithography [25] and the required replica molds were fabricated by photolithography, as described in Figure S1 in the Supplementary Material.

The layout of the microfluidic device was designed with Adobe Illustrator CS6 software. Plastic photomasks for each layer were printed by Newway Photomask, Inc. for fabricating the molds. The mold of parallel micro-gratings (width: 18 μm; depth: 18 μm) was fabricated by deep reactive ion etching with an AZ50XT positive photoresist (AZ Electronic Materials, Somerville, NJ, USA) as the sacrificial layer. On the other hand, molds of the control layer and the flow layer were also fabricated by photolithography of SU−8 photoresist (SU−8 2010, Microchem, Westborough, MA, USA) on planar silicon wafers. Microchannel patterns of the flow layer mold were fabricated with AZ50XT with a height of 20 μm, reflowed at 120 °C for 1 min, while the microchambers and the filtering microstructure around the cell culture region (height: 30 μm) of the mold were aligned with the microchannel patterns and fabricated with an SU-8 2010 photoresist (Microchem, Westborough, MA, USA). All the molds were then silanized by trichloro (1H, 1H, 2H, 2H-perfluoro-octyl) silane (Sigma-Aldrich, St. Louis, MO, USA) for 12 h to facilitate the release of the molded PDMS layers in the later procedures.

Afterwards, PDMS pre-polymer was prepared by mixing the base and the curing agent with a weight ratio of 10:1. The microfluidic device with multiple PDMS layers were fabricated by replica molding of PDMS from the molds using the multiplayer soft lithography as described in Figure S1, with the PDMS applied on the molds with different thickness: 5 mm by pouring on the control layer mold, 35 μm by spin-coating over the flow layer mold, and 1 mm by pouring on the micro-grating mold. All the following bonding processes between PDMS layers were then achieved by air plasma (energy 10 kJ; Harrick plasma cleaner PDC-002, Ithaca, NY, USA). According to the layout of the microfluidic device, as shown in Figure 1a, the PDMS control layer was aligned and bonded onto the flow layer, with holes punched at the gas/liquid inlets and outlets (diameter: 1 mm; WHAWB100073, Sigma-Aldrich, St. Louis, MO, USA), followed by punching a hole (diameter: 6 mm; WHAWB100082, Sigma-Aldrich, St. Louis, MO, USA) at the cell culture region. The PDMS substrate was then bonded on the micro-grating layer, with the micro-grating structures (Figure 1a and Figure S1 in the Supplementary Material) facing the culture chamber. The culturing region was further covered by bonding with another PDMS layer (thickness: 5 mm), with two punched holes as the sample inlet and outlet. Finally, the entire multilayer PDMS substrate was bonded onto a glass slide (Cytoglass, Nanjing, China) for physical support.

### 2.2. Cell Preparation

A human monocytic cell line (THP-1, ATCC TIB-202, Manassas, VA, USA) was cultured in a complete RPMI-1640 culture medium supplemented with 10% fetal bovine serum and 1% penicillin. An immortal human nasopharyngeal epithelial cell line (NP 460) and a nasopharyngeal carcinoma cell line (NPC 43) were developed and donated by the research team of S. W. Tsao, from cell extracts of nasopharyngeal cancer patients [27]. NP 460 cells were cultured in a mixture of 50% complete Eplife medium (Thermo Fisher Scientific, Waltham, MA, USA), 50% complete defined keratinocyte−SFM (Thermo Fisher Scientific, Waltham, MA, USA) with 100 units/mL penicillin and 100 μg/mL streptomycin. NPC 43 cells were maintained in RPMI-1640 (Sigma-Aldrich, St. Louis, MO, USA) added with 10% fetal bovine serum, 4 μM Y27632 dihydrochloride (Alexis), 100 unit/mL penicillin and 100 μg/mL streptomycin. All the cell types were cultured in a 37 °C incubator (HERA cell 150, Thermo Fisher Scientific, Waltham, MA, USA) with a humidified and 5% CO_2_ environment. Additionally, the macrophages were derived from THP−1 cells before experiments. THP−1 cells with a density of 1 × 10^6^ cells/mL was treated with 50 ng/mL of phorbol 12-myristate 13-acetate [28] (PMA; Sigma-Aldrich, St. Louis, MO, USA) for 20 h. The treated cells were then trypsinized (Sigma-Aldrich, St. Louis, MO, USA) for 3 min at room temperature and replaced with fresh media before transferring to the device.

### 2.3. Automated Microscope and Cytokine Quantification

All the microfluidic manipulation for cell culture, monitoring and imaging was operated by an automated microscope platform developed in our laboratory [29]. It mainly includes an inverted fluorescence microscope (IX71, Olympus, Tokyo, Japan) integrated with a microscope camera (Zyla 4.2, Andor Technology Ltd., Belfast, UK) with computer-controlled compressed air supply manifolds and a confining shield mounted on a computer-controlled movable stage of the microscope, offering stable temperature (37 °C), humidity and gas (5% CO_2_) conditions for cell culture (Figure S2 in the Supplementary Material). Cytokine concentrations were quantified using a commercial human inflammatory cytokine kit (Catalog No. 551811, BD Biosciences) in all samples.

### 2.4. Statistics

All experiments were conducted with at least four independent experiments. The *p*-values were calculated using Student’s *t*-test in Excel (Microsoft), with *p* < 0.05 considered as statistically significant.

## 3. Results and Discussion

### 3.1. Device Design and Operation

We have developed an integrated microfluidic immunoassay device, which can implement in-situ and multiplex monitoring of time-lapsed cytokine secretions in an extracellular matrix (ECM) protein-coated topologic environment. This device consists of three main components: a cell culture chamber with parallel microgratings, cytokine detection arrays (four rows and four columns) and an array of active peristaltic mixers (Figure 1a,b). The parallel gratings (each with 18 μm in width and 18 μm in depth) fabricated by polydimethylsiloxane (PDMS) replica molding can mimic the microtopography of pterygoid muscles [23]. The optically transparent cell culture chamber allows the direct observation of cell behaviors and live cell staining under a microscope. Furthermore, it was surrounded by a barrier containing multiple micro-gaps (height: 4 µm; width: 50 µm) to confine cells inside the cell culture chamber, while allowing cell culture media and the secreted cytokines to flow to one of the cytokine detection chambers to achieve the microbeads-based immunoassay for multiple cytokines at different time points. The extracted media were incubated with antibody-conjugated microbeads for capturing cytokines, followed by mixing with phycoerythrin (PE)-conjugated detection antibodies to form fluorescent sandwich complexes for microscopic imaging and cytokine quantification (Figure 1c). In each cytokine detection chamber (volume: 160 nl), a bypass channel (volume: 30 nl) was integrated with a peristaltic mixer, which consisted of three peristaltic microvalves for active pumping and mixing to accelerate the cytokine detection based on the Taylor dispersion effect [30]. The whole assay can be conducted in less than 30 min.

**Figure 2:**
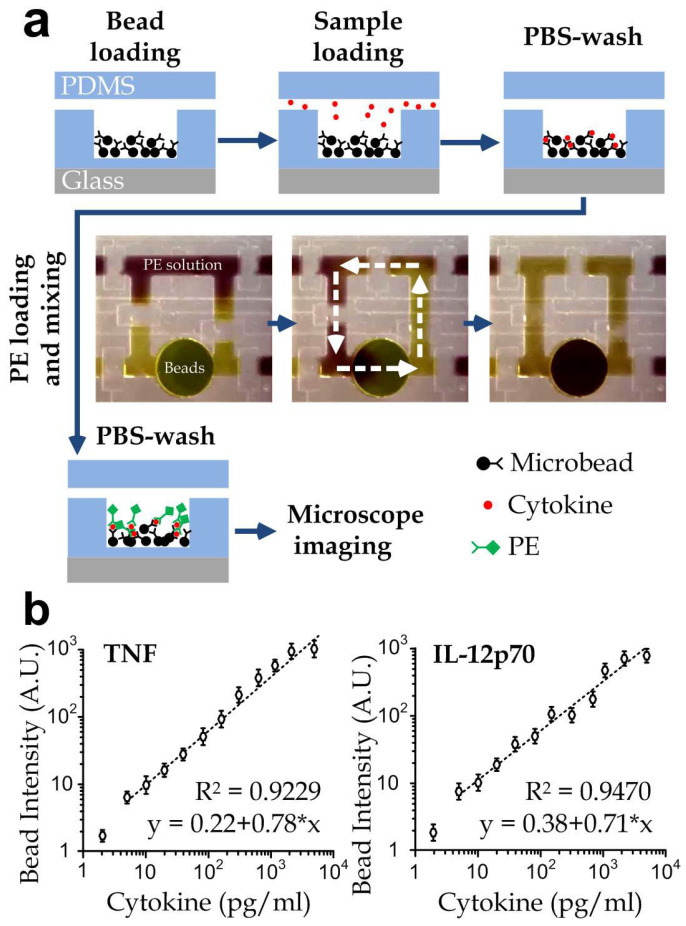
(**a**) Cytokine detection procedures of the integrated microfluidic immunoassay device. Cytokines produced by NPC and immune cells on-chip were transferred from the cell culture region to the cytokine detection arrays, and specifically captured by the pre-loaded antibody conjugated microbeads, followed by washing with PBS buffer. To quantify the cytokine concentrations on-chip, fluorescent phycoerythrin (PE)-conjugated detection antibodies were mixed with the cytokine-binding microbeads using an integrated micromixer (middle panel, representative microscope images), causing the fluorescence change on the microbeads as a readout of binding events. Finally, the fluorescence change was quantified by microscope imaging, and the cytokine concentration was calculated according to the calibration curves. (**b**) The calibration curve of two different cytometric microbeads for TNF and IL12p70 in the microfluidic device by challenging microbeads with different concentrations of cytokines. The data points for 2.4 pg/mL cytokine concentration are not included in the fitting line. Each data point was obtained from the average value of *n* > 100 from 3 repeated measurements. All error bars represent the standard errors.

Notably, we applied 20 μg/mL bovine collagen (Sigma Aldrich, St. Louis, MO, USA) [31] into the culture chamber for 2 h at room temperature before injecting the selected cells, because collagen is a key ECM protein of pterygoid muscles [32]. The nasopharyngeal cells (NP460 or NPC43) and macrophage cells were pre-mixed with a defined population ratio and seeded into the cell culture chamber through the culture inlet and incubated in a microscope incubator with stabilized temperature at 37 °C and 5% CO_2_ to facilitate immune cells secreting cytokines in response to target cells. Simultaneously, the detection microbeads were loaded into the bypass channel aside the cytokine detection chamber by opening the corresponding control valves for defining the access from the ‘Microbeads’ inlet (Figure 1b) to the target detection chamber. We then waited for 10 min for the microbeads sitting on the bottom of the microchamber. The device operation for extracting and quantifying cytokine is shown in Figure 2a and Supplementary video S1. In each measurement, 0.5 μL of the media with secreted cytokines in the culture chamber was extracted from the culture chamber and transported into a detection microchamber with preloaded microbeads for cytokine detection. Meanwhile, fresh media flowed into the culture chamber to supplement the media being extracted. The bypass channel corresponding to the defined cytokine detection chamber was flushed with a PE-conjugated antibody solution, and then the peristaltic mixer was activated for 2 min to mix the PE solution and microbeads (Supplementary video S2). We removed the unconjugated antibody solution by flowing phosphate-buffered saline (PBS) along the detection chamber under gentle driving compressed air with pressure of 0.2 psi for 3 min. The microbeads expressed fluorescence at 647 nm from the bead bodies and the bound cytokine molecules induced fluorescence at 488 nm over the bead surfaces. After imaging the microbeads using an inverted fluorescent microscope, the measured fluorescence intensities at 488 nm were converted to the cytokine concentrations according to the calibration curves (Figure 2b).

To obtain the calibration curves for the selected cytokines, different concentrations (2.4 pg/mL, 4.9 pg/mL, 9.8 pg/mL, 19.5 pg/mL, 39.1 pg/mL, 78.1 pg/mL, 156.3 pg/mL, 312.5 pg/mL, 625 pg/mL, 1250 pg/mL, 2500 pg/mL, 5000 pg/mL) of each cytokine were prepared by standard PBS dilution of the stocking cytokine solution (5 ng/mL). Linear regression with R^2^ > 0.9 was observed between fluorescence intensity (488 nm) and molecular concentrations for each cytokine type. The limit of detection was calculated as ~5 pg/mL for both cytokines based on the concentration, giving a signal equal to the blank signal (Y_0_) plus three standard deviations of the blank (3σ), or 4 pg/mL for TNF and 3 pg/mL for IL12p70 based on 3σ/S, where S is the slope of the calibration curve.

### 3.2. Viability and Cytokine Secretion of Single Cell-Type Cultures

**Figure 3:**
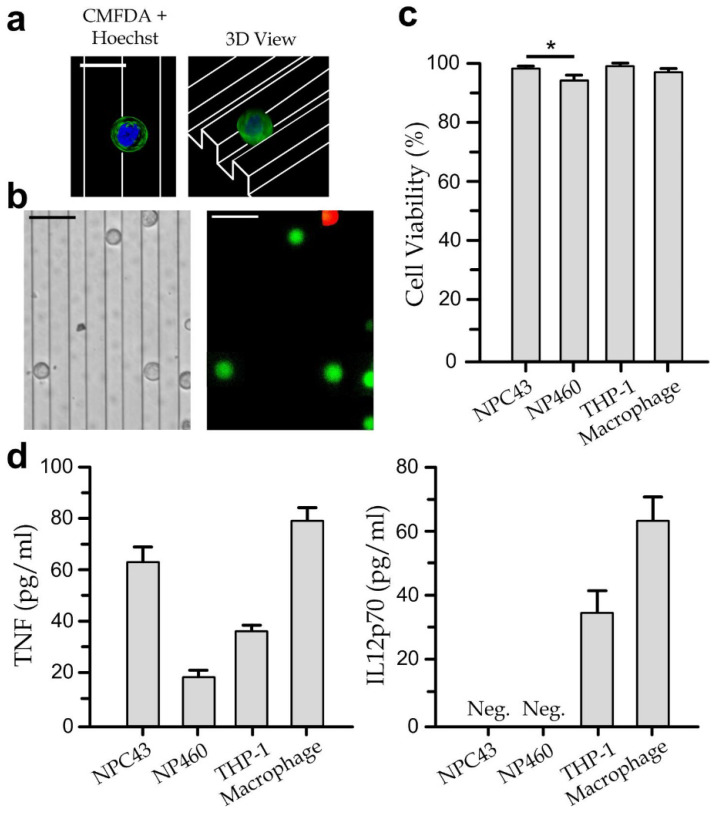
(**a**) Top view (*left* panel) and three-dimensional view (*right* panel) of the reconstructed confocal microscopy images of a NPC43 cell culture on a micro-grating array. Scale bar: 20 µm. (**b**) Brightfield (*left*) and live/dead-stained (*right*) images of NPC43 cultured on micro-gratings for 8 h. Scale bar: 30 µm. (**c**) Viability of NPC43, NP460, THP-1 and THP-1-derived macrophages in the culturing chamber for 8 h. Asterisk represents a *p*-value of <0.05 calculated by Student’s *t*-test. (**d**) Cytokine secretions of mono cultured NPC43/NP460 cells and immune cells on micro-gratings after 8 h of culture. Error bars represent the standard errors.

To verify cell viability in the culture chamber, we applied live/dead-cell staining (L-3224, Thermo Fisher Scientific, Waltham, MA, USA) to the immune cells (THP-1 cells and differentiated macrophages) and nasopharyngeal cells (NP460 and NPC43) growing in the culture chamber at a cell density of 1 × 10^5^ cells/mL. For instance, a stained NPC43 pseudopod attaching on a micro-grating can be captured as shown in Figure 3a, which suggests the good biocompatibility of the on-chip microtopographic environment; and three-dimensional fluorescence micrographs of stained NPC43 cells are shown in Figure 3b, where green cells are live cells and red cells are dead cells. Our results (Figure 3c) indicate that viabilities of NP460, NPC43, THP-1 and macrophages are maintained at >95% for the cells growing in the culture chamber for 24 h.

Additionally, we have also measured the cytokine secretion levels (TNF and IL-12p70) of the single cell-type cultures as summarized in Figure 3d. As described above, the simulated macrophages produce more TNF-α and IL-12 [5,6]; and EBV-infection of the monocytes can cause suppressed IL-12 secretion [8] via the induced MCP-1 expression [7]. Therefore, we selected and quantified TNF and IL-12p70 (an active heterodimer of IL-12) for reflecting the simulated activity and the EBV-infection of the macrophages, respectively. Each of the cell types was seeded in the culture chamber at a density of 1 × 10^5^ cells/mL and incubated for 8 h before the cytokine quantification. In brief, our results show that the unstimulated macrophages can secrete a measurable level of IL-12-p70 (65 pg/mL); whereas relatively larger portions of TNF can be contributed from macrophages (80 pg/mL) and NPC43 cells (62 pg/mL). Such measurements of the single cell-type cultures can determine the baselines of the cytokine levels for the immune-nasopharyngeal cell cocultures.

### 3.3. Cytokine Secretion Dynamics of Immune Cells Co-Cultured with Nasopharyngeal Cells

In the measurements of the cytokine dynamics, immune cells (1 × 10^5^ cells/mL) and nasopharyngeal cells (1 × 10^5^ cells/mL) were cocultured in the culture chamber, with either undifferentiated THP-1 cells or differentiated macrophages applied as the immune cells, and either EBV-positive nasopharyngeal carcinoma (NPC43) cells or EBV-negative nasopharyngeal (NP460) cells applied as the nasopharyngeal cells. Macrophage cells were differentiated from the THP-1 cells by PMA with a concentration of 50 ng/mL for 24 h before the measurements. The cytokine measurements of TNF and IL-12-p70 were performed prior to the culture and at 3, 4, 5, 6, 7 and 8 h of culture. The first three hours of culture could offer a stable environment for cell adaptation.

**Figure 4:**
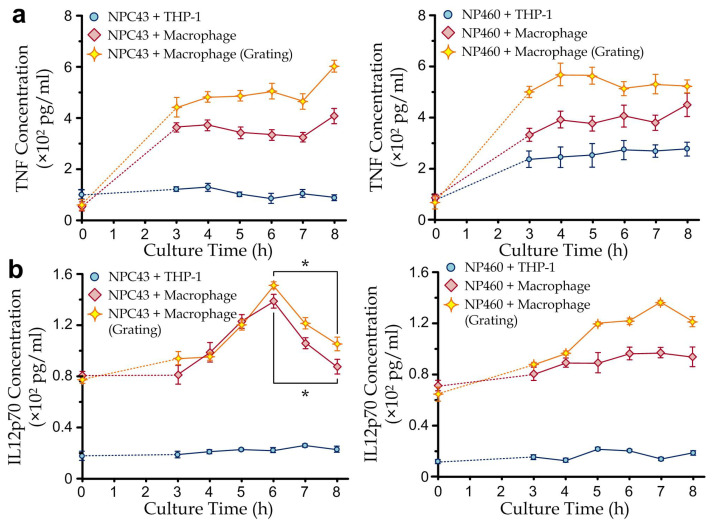
(**a**) The concentration of TNF secreted from cocultured undifferentiated THP-1 and THP-1-differentiated macrophages with NPC43 cells or NP460 cells, with and without a parallel grating array. (**b**) The concentration of IL-12p70 secreted from THP-1 and macrophages co-cultured with NPC43 cells or NP460 cells, with and without parallel grating array (*n* = 4 for each point). Error bars are the standard errors in all plots. Asterisk represents a *p*-value of <0.05.

In the measurements as shown in Figure 4a, we have considered both NPC43 and NP460 in three conditions for different purposes: (1) cocultured with undifferentiated THP-1 on a flat surface as a control case, (2) cocultured with THP-1-differentiated macrophages on a flat surface for estimating the NPC43/NP460-stimulated cytokine secretion, and (3) cocultured with THP-1-derived macrophages on microgratings for revealing the topography-induced cytokine responses. 

For the TNF measurements, our results show that the differentiated macrophages secrete a much higher level of the pro-inflammatory cytokine TNF than the undifferentiated THP-1 monocytic cells for both cocultured cases (NPC43 and NP460). The TNF secretions of the cell cocultures on microgratings have an increment of ~20% on average, compared to the cells on flat surfaces, implicating an underlying mechanism related to the microtopographic factors (Figure 4a). Nevertheless, the cytokine secretions of both macrophages and monocytic cells cocultured with NPC43 are less than those cocultured with NP460, suggesting that NPC43 may suppress the TNF secretion of immune cells. One possible explanation is that EBV can suppress TNF-α synthesis from lipopolysaccharide-treated monocytes at both protein and transcriptional levels as reported previously [33].

For the IL-12p70 measurements, it is noteworthy that a distinct secretion profile of IL-12p70 was observed compared to that of TNF (Figure 4b). Though similar trends as the TNF secretion cases have been shown that the IL-12p70 levels are, in general, higher (1) in the macrophages cocultures than in the monocyte cocultures and (2) on the microgratings than on the flat surface for the macrophage cocultures; the coculture with NPC43 does not necessarily induce higher IL-12p70 levels than the coculture with NP460. Furthermore, IL-12p70 secretions of the macrophage-NPC43 cocultures have increasing cytokine levels in the initial stage (0–6 h), reaching the maximum levels at ~6 h and gradually decreasing afterward. Notably, the coefficient of variation (CV), the ratio of the standard deviation to the mean, was investigated for repeatability, which was less than 15% for all data.

These results reveal some insightful observations. Interestingly, the macrophage-NPC43 cocultures exhibit suppressed IL-12p70 expressions after 6 h of coculture, implicating an underlying related mechanism between macrophages and nasopharyngeal cancer cells. This agrees with previous clinical studies that nasopharyngeal carcinoma patients have a reduced level of IL-12p70 in their serum [9]. This indicates the suppression of IL-12 secretion, which can be caused by the induced MCP-1 expression of the EBV-infected macrophages [7]. EBV-infection of microphages can also promote polarization to the M2 macrophages [34], leaving a smaller portion of IL-12p70 secreting M1 macrophages [35]. On the other hand, our results show that the microgratings as a microtopographic factor can induce the cytokine secretions of TNF and IL-12p70 on top of the molecular interactions between the immune and nasopharyngeal cells. One possible explanation is that cells adhering to the parallel gratings have different cell behaviors, e.g., morphology and migration [23], which can then affect the immune-cancer cell interaction to some extents. Therefore, it is worthwhile to further apply the reported microfluidic immunoassay to investigate the cell-microenvironment dependency through the simultaneous monitoring of cell behaviors during the coculture periods.

### 3.4. Cell Migration

**Figure 5:**
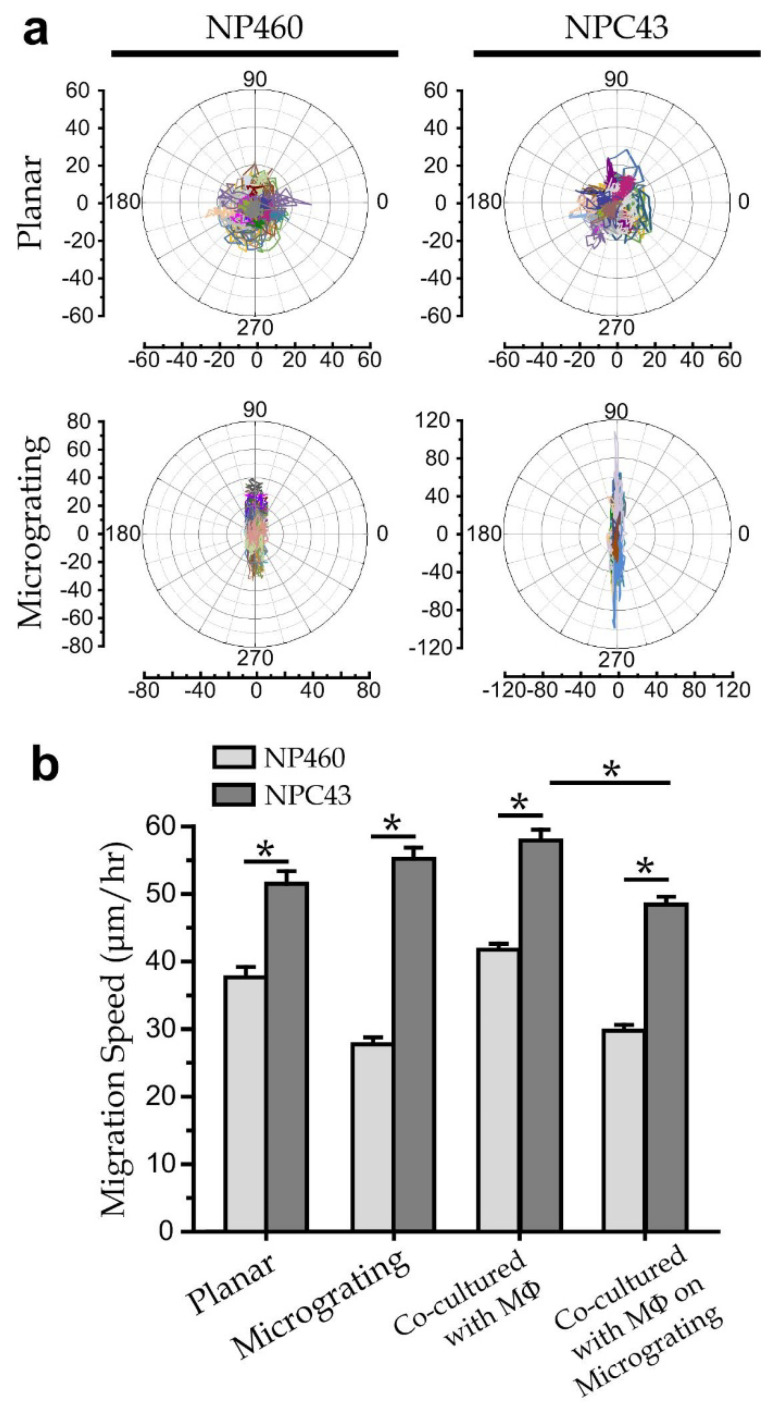
(**a**) Migration trajectories of single NP460 and NPC43 cells on flat/grating substrates. (**b**) Migration speed of single NP460, single NPC43 and those co-cultured with THP-1 derived macrophages on platforms with/without grating. *n* > 35 for all cases. Error bars represent the standard errors. Asterisk represents a *p*-value of <0.05.

We further investigated the migration behaviors of NP460 and NPC43 single cell co-cultured with or without THP-1 derived macrophages on grating platforms. We seeded NP460/MPC43 cells at a density of 5 × 10^2^ cells/cm^2^ and each of the co-cultured cells at a density of 2.5 × 10^2^ cells/cm^2^, followed by culturing the cells for 8 h and monitoring their migration under a microscope. Migration trajectories of NP460 and NPC43 cells growing on the planar/microgratings substrates (along 90°/270°) are shown in Figure 5a. Clearly, the cells on planar surfaces display a random migration trajectory, whereas the cells on microgratings migrate with a direction along the microgratings.

Our results (Figure 5b) further indicate that NPC43 cells migrate faster than NP460 cells on both planar and micrograting surfaces. NPC43 cells can migrate even faster when they are cultured on micro-grating substrates than on planar substrates. On the other hand, NP460 cells migrate slower on microgratings than they do on planar substrates, which suggests the different responses of NP460 and NPC43 cells upon the microgratings topography. Furthermore, the co-cultures of macrophages with NP460/NPC43 cells on planar substrates induce faster cell migration, suggesting that molecular secretions of macrophages can promote cell migration. In fact, it has been reported that the MCP-1 secreted by macrophages can promote cell migration [36]. Furthermore, our results indicate that >5% of macrophages and NPC43 cells adhered together on microgratings without further migration, whereas the other cells appearing as single cells without noticeable cell-cell contact can still maintain at a faster migration speed. Interestingly, the cytokine and migration measurements exhibit that nasopharyngeal cancer cells can stay on the microgratings with suppressed IL-12 secretion of the contacting macrophages, supporting the higher tendency of nasopharyngeal cancer spreading to the grating-like pterygoid muscles [37]. Together, the microgratings can affect cell migration behaviors and possibly the intracellular interactions of nasopharyngeal cells (NP460 and NPC43) and immune cells. For example, it is worthwhile to further examine the correlation between the direct NPC43/macrophage contact and the suppressed IL-12 secretion, and the underlying mechanism.

There are several limitations in the current study. For instance, the effects of interference components such as cell debris in detection samples would be eliminated by integrating a porous membrane filter [38] for sample pretreatments before cytokine detection. Integrating the current microfluidic immunoassay with non-washing cytokine detection strategy such as AlphaLISA [39] may further improve the detection sensitivity and specificity. Simultaneous monitoring of cell behaviors and highly multiplex cytokine detection during the coculture periods using the developed immunoassay would provide valuable insights into the comprehensive and dynamic immune status in solid tumors and during inflammatory states that result in heterogeneous tumor microenvironmental features for precision medicine. Moreover, standardized and automated fabrication setup such as cost-efficient and multilayer PDMS aligner [40] should be further developed for large-scale and high throughput fabrication of the developed microfluidic immunoassay.

## 4. Conclusions

In conclusion, we have reported a multifunctional microfluidic immunoassay by integrating microtopographic cell-culture substrates with a microbeads-based immunofluorescence assay that enables parallel detection of different immune biomarkers and intercellular behaviors in a rapid, sensitive, and easy-to-implement manner. The developed assay exhibits the advantages of the simultaneous investigation of different cytokines and cell migration behaviors on flat/grating ECM substrates, requiring a low-volume sample (0.5 μL) and short assay time (30 min) but a sensitive performance in a wide range of cytokine concentrations (5–5000 pg/mL). Secretions of TNF and IL-12p70 were successfully monitored throughout the co-culture period to evaluate the different immunological states of undifferentiated THP-1 monocytic cells or PMA-differentiated THP-1 macrophages co-cultured with immortal cells NP460/NPC43 on flat and micrograting surfaces. We believe that the reported immunoassay is a promising approach to allow continuous, broad-range and precise on-chip characterization of cytokine and intercellular interactions on different topographical substrates, and thus provides clinical significance for early tumor diagnosis and treatment.

## Data Availability

Not applicable.

## Supplementary Materials

The following supporting information can be downloaded at: www.mdpi.com/article/10.3390/bios12110963/s1, Figure S1: Fabrication process of the integrated microfluidic immunoassay; Figure S2. A microscope system integrated with a confining shield (a) and computer-controlled compressed air supply manifolds (b); Video S1: Device operation for extracting culture media from the cell culture chamber; Video S2: Cytokine detection procedures in one detection unit integrated with an active micromixer.
