# Supplementary material for: A Microfluidic Platform Revealing Interactions between Leukocytes and Cancer Cells on Topographic Micropatterns
Source: Biosensors (Basel). 2022 Nov 2;12(11):963. doi: 10.3390/bios12110963 (PMC9687854; doi:10.3390/bios12110963)
Supplement: Supplementary file 1 [file biosensors-12-00963-s001.zip › Supplementary materials.pdf]

Supplementary materials

A microfluidic platform revealing interactions between leukocytes and cancer cells on topographic micropatterns

Xin Cui[1,#,*], Lelin Liu[2,3,#], Jiyu Li[2,#], Yi Liu[2], Ya Liu[4], Dinglong Hu[4], Ruolin Zhang[5,6] Siping Huang[2], Zhongning Jiang[2], Yuchao Wang[2], Yun Qu[2], Stella W. Pang[6,7], and Raymond H. W. Lam[2,7,8,9*]

[1] Key Laboratory of Biomaterials of Guangdong Higher Education Institutes, Department of Biomedical Engineering, Jinan University, Guangzhou, China

[2] Department of Biomedical Engineering, City University of Hong Kong, Hong Kong, China

[3] Research Center of Biological Computation, Zhejiang Laboratory, Hangzhou 311100, China

[4] BGI-Shenzhen, Shenzhen 518083, Guangdong, China

[5] Department of Electrical Engineering, City University of Hong Kong, Hong Kong, China

[6] Department of Biomedical Engineering, The Hong Kong Polytechnic University, Hong Kong, China

[7] Centre for Biosystems, Neuroscience, and Nanotechnology, City University of Hong Kong, Hong Kong, China

[8] Centre for Robotics and Automation, City University of Hong Kong, Hong Kong, China

[9] City University of Hong Kong Shenzhen Research Institute, Shenzhen, China

# Authors equally contributed to this work.

* Correspondence should be addressed to X. Cui (email: cx2019@jnu.edu.cn; Tel: +86-8522-0469) and R.H.W. Lam (email: rhwlam@cityu.edu.hk; Tel: +852-3442-8577; Fax: +852-3442-0172)

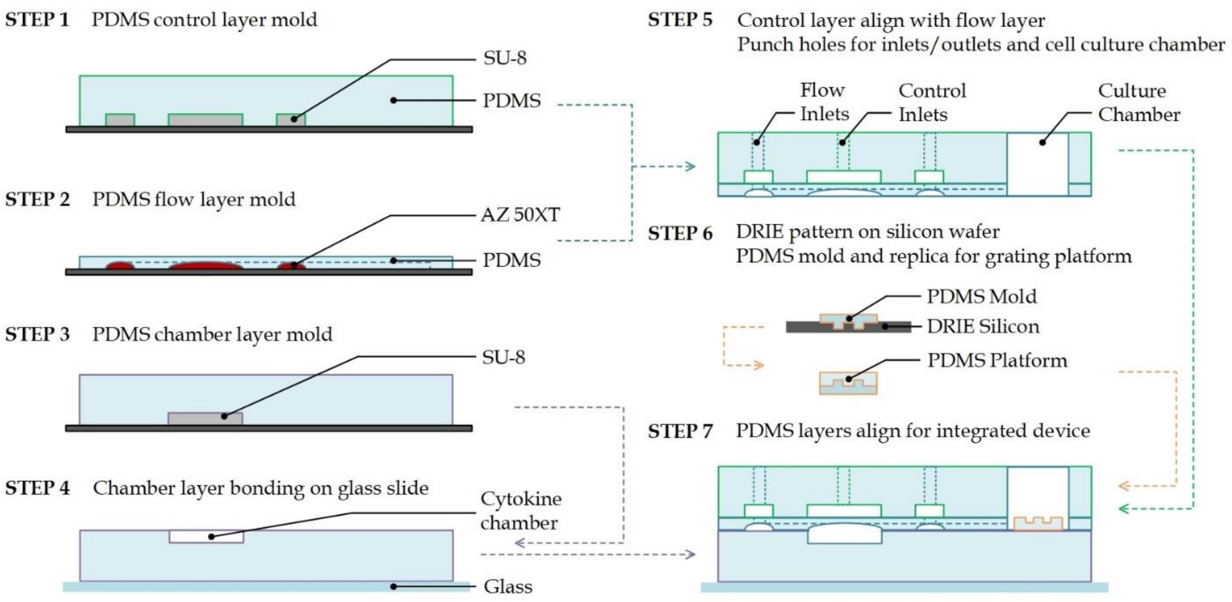

Supplementary Figure S1. Fabrication process of the integrated microfluidic immunoassay.

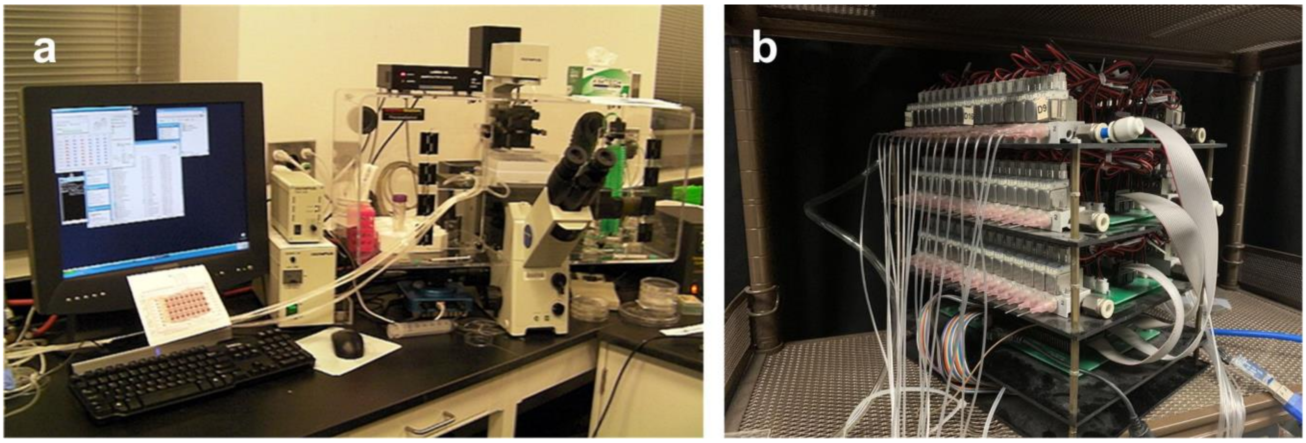

Supplementary Figure S2. A microscope system integrated with a confining shield (**a**) and computer-controlled compressed air supply manifolds (**b**).

Supplementary video S1. Device operation for extracting culture media from the cell culture chamber. *Red* circle highlights immune cells (THP-1) trapped in cell culture chamber.

Supplementary video S2. Cytokine detection procedures in one detection unit integrated with an active micromixer. Different colour dyes are used to represent PE-conjugated antibody solution (*purple*) and antibody-conjugated microbeads (*green*).
